# Gender-specific differences in severely injured patients between 2002 and 2011: data analysis with matched-pair analysis

**DOI:** 10.1186/cc13132

**Published:** 2013-11-29

**Authors:** Carsten Schoeneberg, Max Daniel Kauther, Bjoern Hussmann, Judith Keitel, Daniel Schmitz, Sven Lendemans

**Affiliations:** 1 Department of Trauma Surgery, University Hospital Essen, University Duisburg-Essen, Hufelandstraße 55, 45147 Essen, Germany

## Abstract

**Introduction:**

Previous studies reported divergent results concerning the effect of gender on patient outcome after severe injury. Results suggest that women have better outcomes because they have lower rates of sepsis and multi-organ failure. The objective of this analysis was to study gender differences in a Level 1 trauma center in Germany.

**Methods:**

Patients who were admitted to hospital between 2002 and 2011 with an Injury Severity Score (ISS) ≥16 were included. Data were collected from the Trauma Registry of the German Society for Trauma Surgery and from hospital records. The effects of gender on a variety of parameters were investigated. To eliminate the influence of differences in ISS, an analysis of groups with similar ISS was performed. Also, a matched-pair analysis of 422 patients was performed.

**Results:**

A total of 962 patients met the inclusion criteria. The mortality rate was lower in male patients (25.4% versus 36.59%). Female patients had more severe head injuries, received less fluid volume and had a lower rate of sepsis. Men were more frequently involved in motorcycle accidents and sustained more penetrating trauma. Women were more frequently involved in pedestrian accidents and sustained more falls from under 3 m. The effects of gender were reduced when the data were analyzed by matching ISS. The mortality rate was significantly different in the ISS 26 to 35 group but in mostly all groups, the mortality rate was higher in women. In the matched-pair analysis, the rate of sepsis and the length of the ICU stay were significantly lower in women and the mortality rate showed no significant difference (28.1% for male patients versus 33.01% for female patients). Women died after an average of 5.22 days, and men died after an average of 9.02 days.

**Conclusions:**

Gender-based differences in patient outcome after severe trauma were observed in this study. Women are more likely to die in the first days after trauma. Upon extended hospital stay, women had a better survival rate because they had a lower rate of sepsis. No significant differences in mortality rate could be found, but there was a trend towards a higher rate in female patients.

## Introduction

Trauma is the third most common cause of death in industrialized countries and the most frequent cause of death in people younger than 44 years old [1]. Previous studies have suggested that gender influences patient outcome after severe trauma. However, these studies have reported divergent effects of gender. Some studies reported better outcomes for male patients [2,3], but other studies suggested that female patients had better outcomes after severe trauma [4,5]. Also no differences in mortality have previously been reported [6-8]. Despite the divergent results of previous studies, the literature suggests that female patients may have better outcomes and survival rates after severe trauma.

Choudhry and colleagues observed that male and female patients respond differently after hemorrhagic shock and trauma [9]. These differences were further evaluated in experimental animal studies, and the results demonstrated that sex hormones were responsible for these gender-based differences [10]. However, clinical evaluation of these experimental findings generated inconsistent results [11,12].

Frink and colleagues observed a significant reduction in multi-organ failure (MOF) and sepsis in female patients compared with similarly aged male patients [6]. These results were associated with a lower serum level of inflammatory cytokines in female patients after severe trauma. Majetschak and colleagues reported similar results. They observed a higher rate of sepsis in male patients, caused by a difference in leukocyte function [13]. Sperry and colleagues observed a reduced rate of MOF and sepsis in female patients. The authors of this study suggested that factors other than sex hormones are responsible for the observed differences between genders [14].

Previous studies have not reported significant gender-based differences in the time of death after trauma. The main objective of this analysis was to characterize gender-based differences in patient outcome at a Level 1 trauma center in Germany. To avoid age-dependent, trauma-dependent, or severity-dependent differences, a matched-pair analysis was also performed.

## Materials and methods

This study analyzed data collected for the Trauma Registry of the German Society for Trauma Surgery (DGU). This registry collected data prospectively from collaborating trauma centers. The data from the Trauma Registry of the DGU have received full approval from the Ethics Committee of the University of Witten/Herdecke in Cologne, Germany.

The analysis used data from a Level 1 trauma center, which is one of the largest trauma centers in Germany. As the trauma registry of the DGU is an anonymous register, the Institution Review Board waived the need for patient consent.

Patients were selected according to the following criteria: primary admission to the hospital occurred following trauma; the patient’s Injury Severity Score (ISS) was ≥16; and admission occurred between July 2002 and December 2011.

The following items were collected for each patient: scales – ISS [15], Abbreviated Injury Scale (AIS) [16], New ISS [17], Glasgow Coma Scale [18], Revised Trauma Score [19], Revised Injury Severity Classification (RISC) [20], Trauma and Injury Severity Score; general patient information – age, gender, systolic blood pressure at the accident scene, heart rate at the accident scene, length of ICU stay, length of hospital stay, count of performed surgery, administered fluid volume, MOF, sepsis, type of injury (penetrating vs. blunt), suicide; laboratory test values – first hemoglobin (Hb) value, initial number of platelets, partial thromboplastin time, prothrombin time; length of relevant periods – time from admission to cranial computed tomography, time from admission to whole-body computed tomography, time in trauma room, time from admission to operating room, preclinical rescue time; and interventions – intubation, resuscitation, or thoracic drainage by emergency physician at the accident scene and intubation, resuscitation, or thoracic drainage in the trauma room.

To evaluate gender-based differences, male and female patients were matched according to the following criteria: pattern of injury for six body regions (head, thorax, abdomen, face, skin, and extremities, including the pelvis), where matching criteria were AIS severity ≥3 or <3 points; age, in which patients were divided into five subgroups (0 to 7 years, 8 to 15 years, 16 to 54 years, 55 to 69 years, and ≥70 years); and ISS, in which patients were divided into several subgroups (16 to 25, 26 to 35, 36 to 45, 46 to 55, 56 to 65, and 66 to 75).

Data were analyzed using the Statistical Package for the Social Sciences (version 21; SPSS: An IBM Company; Chicago, IL, USA). Incidences are represented as percentages, and measured values are represented as means and standard deviations. Differences between genders were evaluated using the chi-squared test for categorical variables and the *t* test for continuous variables. When performing the *t* test, a Levene test was also performed. In cases of variance heterogeneity, the Welch test was used instead of the *t* test. When an obvious deviation from normality was detected, continuous variables were tested with a nonparametric rank test (Mann–Whitney test). Differences were considered statistically significant when *P* <0.05.

## Results

**Figure 1:**
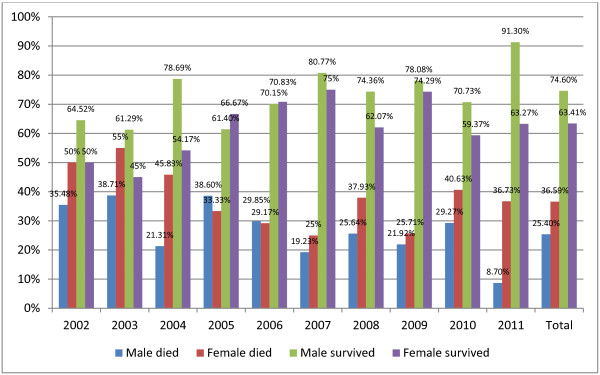
Annual mortality rate divided by gender.

Between July 2002 and December 2011, a total of 962 patients were admitted to the hospital and met the inclusion criteria. Of these patients, 70.89% were male, and the overall mortality rate was 28.7% (276 patients). The mean ISS was 29.81, the mean Glasgow Coma Scale was 9.42, and the mean age was 46.04 years. The annual gender-based mortality rate is presented in Figure 1.

The overall mortality rate and most of the annual mortality rates were lower in male patients. The overall mortality rates were 25.4% in male patients and 36.59% in female patients. This difference was statistically significant (*P* = 0.001).

To further investigate this difference, an analysis of different parameters was performed. Table 1 presents the observed gender-based differences and the corresponding significance values.

**Table 1:** Gender-based differences

	**Male**	**Female**	***P *** **value**
Patients (*n*)	684	278	
Glasgow Coma Scale	9.73 ± 5.04	8.6 ± 4.93	0.002*
Revised Trauma Score	5.94 ± 2.28	5.68 ± 2.17	0.045*
Injury Severity Score	29.52 ± 14.22	30.58 ± 14.53	0.32
New Injury Severity Score	36.45 ± 16.34	37.66 ± 16.8	0.36
Trauma and Injury Severity Score	0.71 ± 0.35	0.62 ± 0.36	<0.001*
Revised Injury Severity Classification	25.33 ± 32.18	34.93 ± 32.94	<0.001*
AIS head	2.85 ± 2.07	3.46 ± 1.88	<0.001*
AIS face	0.47 ± 0.91	0.49 ± 0.96	0.87
AIS thorax	2.04 ± 1.88	1.69 ± 1.92	0.011*
AIS abdomen	0.8 ± 1.43	0.82 ± 1.47	0.9
AIS extremities	1.34 ± 1.51	1.46 ± 1.59	0.29
AIS skin	0.45 ± 0.79	0.42 ± 0.7	0.57
Age	43.5 ± 20.89	52.06 ± 25.54	<0.001*
blood pressure systolic	119.97 ± 42.26	122.80 ± 39.52	0.39
Heart rate	91.27 ± 29.35	90.18 ± 28.44	0.61
Hemoglobin (g/dl)	11.78 ± 2.74	10.66 ± 2.39	<0.001*
Platelets gigaparticle per litre (gpt/l)	195.37 ± 85.20	202.91 ± 73.42	0.044*
Partial thromboplastin time (seconds)	33.23 ± 24.63	30.68 ± 16.95	0.127
Prothrombin time (%)	82.71 ± 26.45	82.49 ± 27.75	0.915
ICU stay (days)	14.08 ± 19.08	10.48 ± 15.06	0.007*
Hospital stay (days)	21.45 ± 24.03	16.79 ± 20.04	0.002*
Number of surgery	2.79 ± 4.80	2.08 ± 2.71	0.03*
Time from admission to CCT (minutes)	32.43 ± 15.84	33.08 ± 17.21	0.659
Time from admission to whole-body CT (minutes)	35.04 ± 17.08	34.66 ± 17.63	0.82
Pre-hospital volume (ml)	1,207.37 ± 1,014.62	1,026.09 ± 805.57	0.026*
Trauma room volume (ml)	1,725.90 ± 1,802.89	1,572.70 ± 1,584.00	0.25
Total volume (ml)	3,004.09 ± 2,434.72	2,609.81 ± 1,990.07	0.012*
Time in trauma room (minutes)	56.38 ± 28.20	54.19 ± 25.90	0.29
Time until surgery (minutes)	63.84 ± 31.79	59.86 ± 27.81	0.321
Preclinical rescue time (minutes)	49.19 ± 24.80	42.14 ± 18.51	0.005*
Rate of whole-body CT	63.11	62.86	0.94
Multi-organ failure	33.82	34.66	0.81
Sepsis	21.68	15.12	0.026*
Intubation at the accident scene	63.35	64.73	0.69
Resuscitation at the accident scene	8.68	6.34	0.24
Thoracic drainage at the accident scene	8.09	2.61	0.002*
Intubation in trauma room	43.1	42.05	0.77
Resuscitation in trauma room	7.56	9.23	0.4
Thoracic drainage in trauma room	17.18	16.47	0.8
Surgery needed	72.95	70.86	0.51

Data presented as mean ± standard deviation or percentage. AIS, Abbreviated Injury Scale; CCT, cranial computed tomography; CT, computed tomography. *Differences are significant.

The gender groups differed very little in ISS and New ISS. Significant differences were observed in the Glasgow Coma Scale, Revised Trauma Score, Trauma and Injury Severity Score, and RISC. Female patients sustained severe head injuries more frequently (AIS head 3.46 vs. 2.85), and male patients sustained severe thorax injuries more frequently (AIS thorax 2.04 vs. 1.69). On average, female patients were 9 years older than male patients. In initial laboratory tests, the Hb level was significantly lower in female patients. Women had a shorter stay in the ICU (10.48 days vs. 14.08 days) and a shorter hospital stay (16.79 days vs. 21.45 days). At the accident scene, female patients received a lower fluid volume (1,026.09 ml vs. 1,207.37 ml) and required the placement of thoracic drains less frequently. Treatment in the trauma room was similar in both groups, and no significant differences were observed. Male patients required surgery more frequently (2.79 surgeries/stay vs. 2.08 surgeries/stay) and developed sepsis more frequently during their hospital stay (21.68% vs. 15.12%). The rate of MOF was similar in both groups.

Male patients were involved in traffic accidents with motorcycles significantly more often (14.8% vs. 1.4%), and they were victims of penetrating trauma more often (11.84% vs. 3.61%). The main cause of injury in women was traffic accidents as a pedestrian (22.3% vs. 9.3%). Women also sustained falls from <3 m more often (27.0% vs. 13.7%). The percentages for specific causes of injuries are presented in Table 2.

**Table 2:** Gender-based differences in the cause of injuries

**Cause of injury**	**Male (%)**	**Female (%)**	***P *** **value**
Suicide	11.5	9.4	0.32
Traffic accident, car	14.2	11.5	0.26
Traffic accident, motorcycle	14.8	1.4	<0.001*
Traffic accident, bicycle	6.0	5.0	0.55
Traffic accident, pedestrian	9.3	22.3	<0.001*
Fall >3 m	21.4	19.1	0.41
Fall <3 m	13.7	27.0	<0.001*
Suspected violent crime	5.0	4.0	0.49
Penetrating trauma	11.84	3.61	<0.001*

*Significant difference.

Gender-based differences were also analyzed for groups of patients that were divided based on the severity of injury as indicated by ISS. Starting with an ISS of 16, a total of six groups were formed, and each group included patients with a range of 10 ISS points. Table 3 shows the significant differences.

**Table 3:** Significant gender-based differences in groups divided by Injury Severity Score

**ISS**		**Male**	**Female**	***P *** **value**
16 to 25	Glasgow Coma Scale	11.15 ± 4.55	9.76 ± 4.86	0.005
	Revised Injury Severity Classification	14.91 ± 24.18	24.42 ± 28.07	<0.001
	AIS head	2.45 ± 1.95	3.15 ± 1.84	<0.001
	AIS thorax	1.43 ± 1.65	0.89 ± 1.44	<0.001
	AIS skin	0.47 ± 0.82	0.27 ± 0.55	0.002
	Age	43.87 ± 21.37	53.53 ± 27.01	<0.001
	Hemoglobin (g/dl)	12.40 ± 2.40	11.28 ± 2.00	<0.001
	Number of surgery	2.24 ± 4.91	1.42 ± 2.11	0.014
	Pre-hospital volume (ml)	1,063.19 ± 932.57	839.04 ± 721.34	0.014
	Trauma room volume (ml)	1,420.70 ± 1,365.01	1,233.03 ± 1,216.45	0.37
	Total volume (ml)	2,514.12 ± 1,965.05	2,140.85 ± 1,704.13	0.005
	Preclinical rescue time (minutes)	47.66 ± 20.77	39.98 ± 17.50	0.01
	Penetrating Trauma	10.74	4.10	0.017
	Mortality rate	15.66	21.38	0.12
26 to 35	Revised Injury Severity Classification	24.00 ± 29.54	34.21 ± 31.17	0.012
	AIS head	2.87 ± 2.05	3.76 ± 1.70	0.001
	AIS thorax	2.35 ± 1.83	1.32 ± 1.79	<0.001
	AIS skin	0.47 ± 0.72	0.71 ± 0.66	0.018
	Age	43.67 ± 20.28	53.56 ± 23.82	0.004
	Hemoglobin (g/dl)	11.53 ± 2.46	10.61 ± 2.43	0.005
	Resuscitation at the accident scene	7.23	0	0.032
	Thoracic drainage at the accident scene	9.10	0	0.016
	Thoracic drainage in trauma room	18.93	5.10	0.011
	Sepsis	32.12	13.11	0.004
	Suicide	5.71	14.29	0.031
	Mortality rate	25.0	42.86	0.008
36 to 45	Trauma and Injury Severity Score	0.55 ± 0.33	0.33 ± 0.31	0.008
	Age	41.40 ± 18.95	54.77 ± 21.93	0.003
	Hemoglobin (g/dl)	11.03 ± 3.48	9.68 ± 2.37	0.008
	Platelets gigaparticle per litre (gpt/l)	175.48 ± 70.96	217.85 ± 75.56	0.011
	Time from admission to CCT (min)	34.84 ± 17.46	44.91 ± 26.48	0.047
	Time from admission to whole-body CT	36.47 ± 18.86	47.57 ± 26.48	0.005
	Suicide	7.8	29.03	0.004
	Mortality rate	27.27	46.67	0.055
46 to 55	Age	40.94 ± 21.18	51.80 ± 19.44	0.14
	Mortality rate	55.56	53.33	0.9
56 to 65	Age	39.80 ± 22.14	37.29 ± 32.56	0.85
	Mortality rate	60.0	85.71	0.25
66 to 75	Age	41.40 ± 18.95	54.77 ± 21.93	0.125
	Glasgow Coma Scale	4.33 ± 3.55	5.25 ± 2.98	0.047
	AIS abdomen	0.68 ± 1.7	3.06 ± 2.21	0.001
	AIS extremities	1.10 ± 1.76	2.81 ± 2.01	0.025
	Penetrating trauma	35.9	6.25	0.047
	Sepsis	0	20.0	0.019
	Mortality rate	90	93.75	0.657

Data presented as mean ± standard deviation or percentage. AIS, Abbreviated Injury Scale; CCT, cranial computed tomography; CT, computed tomography; ISS, Injury Severity Score.

**Figure 2:**
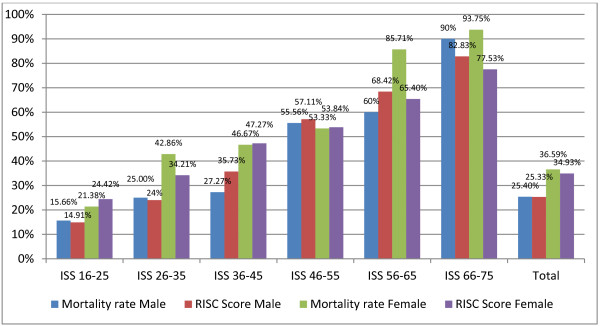
**Gender-based mortality and revised injury severity classification in different injury severity score groups.** ISS, Injury Severity Score; RISC, Revised Injury Severity Classification.

With rising ISS, significant differences between genders decreased. The severity of injury within individual body regions between groups was approximately equal. For patients with ISS >36, with the exception of the ISS 66 to 75 group, no statistically significant differences were observed between genders. The average patient age was also similar. A significant difference in mortality rates between genders was observed only in the ISS 26 to 35 group. But in all groups, except the ISS 46 to 55 group, there was a trend for a higher mortality rate in females. The ratio of the mortality rate and the gender-based RISC are shown in Figure 2.

The mortality rate was higher in women than in men in nearly every ISS group. A comparison of the gender-based RISC with the mortality rate revealed that in women the mortality rate was more likely to exceed the RISC. In men, the mortality rate was more likely to be equal to or below the RISC. This difference was particularly noticeable in the ISS 56 to 65 group and the ISS 66 to 75 group. In these groups, the mortality rates in female patients were 85.71% and 93.75%, respectively, and the RISC scores were 65.4% and 77.53%, respectively.

To minimize the influence of additional factors, such as age, ISS, AIS and cause of trauma (blunt or penetrating trauma), a matched-pair analysis was performed. A total of 422 patients were included. Subsequent analysis of the criteria for matching criteria demonstrated nearly identical mean values for women and men (Table 4).

**Table 4:** Mean values of the matching criteria

	**Male**	**Female**	***P *** **value**
Patients (*n*)	211	211	
Injury Severity Score	26.91 ± 11.03	27.59 ± 11.71	0.54
AIS head	3.37 ± 1.8	3.48 ± 1.8	0.53
AIS face	0.40 ± 0.81	0.39 ± 0.83	0.91
AIS thorax	1.57 ± 1.85	1.53 ± 1.86	0.81
AIS abdomen	0.62 ± 1.24	0.63 ± 1.28	0.91
AIS extremities	1.24 ± 1.45	1.23 ± 1.45	0.92
AIS skin	0.36 ± 0.64	0.37 ± 0.59	0.81
Age	49.89 ± 23.13	50.28 ± 24.41	0.87
Penetrating trauma	2.84	2.84	

Data presented as mean ± standard deviation or percentage. AIS, Abbreviated Injury Scale.

**Figure 3:**
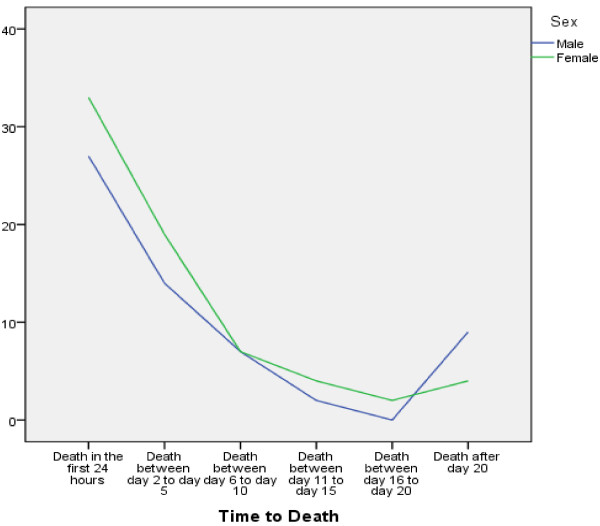
Gender difference in time to death.

When comparing genders with a matched-pair analysis, the laboratory Hb values and platelet numbers were significantly different. On average, women were treated for a shorter period in the ICU and in the hospital. The total time in the emergency room was shorter for women. In men, thoracic drains were placed by the emergency physician more frequently. Women had a lower rate of sepsis. The mortality rate was 28.1% in men and 33.01% in women, but this difference was not significant (*P* = 0.27). Women died after an average of 5.22 days, and men died after an average of 9.02 days. However, this difference was not significant (*P* = 0.22) (Figure 3). Gender-based differences in the matched-pair analysis are presented in Table 5.

**Table 5:** Differences between males and females: results of the matched-pair analysis

	**Male**	**Female**	***P *** **value**
Patients (*n*)	211	211	
Glasgow Coma Scale	9.51 ± 4.85	8.84 ± 4.97	0.056
Revised Trauma Score	5.94 ± 2.11	5.79 ± 2.05	0.24
New Injury Severity Score	34.40 ± 14.61	35.00 ± 15.63	0.065
Trauma and Injury Severity Score	0.69 ± 0.35	0.67 ± 0.34	0.62
Revised Injury Severity Classification	28.34 ± 32.28	29.04 ± 30.06	0.82
blood pressure systolic	129.43 ± 42.27	125.57 ± 35.99	0.063
Heart rate	92.19 ± 26.90	91.46 ± 26.77	0.47
Hemoglobin (g/dl)	12.01 ± 2.78	10.99 ± 2.12	<0.0001*
Platelets gigaparticle per litre (gpt/l)	189.30 ± 69.02	203.68 ± 73.08	0.035*
Partial thromboplastin time (seconds)	31.38 ± 20.86	29.88 ± 16.39	0.67
Prothrombin time (%)	81.44 ± 27.93	84.99 ± 27.04	0.76
ICU stay (days)	14.94 ± 21.44	10.05 ± 13.85	0.019*
Hospital stay (days)	21.86 ± 25.65	16.12 ± 18.83	0.01*
Number of surgeries	2.36 ± 3.64	1.89 ± 2.48	0.84
Time from admission to CCT (minutes)	32.75 ± 14.48	30.69 ± 13.19	0.88
Time from admission to whole-body CT (minutes)	34.99 ± 15.79	32.08 ± 13.82	0.62
Pre-hospital volume (ml)	1,120.81 ± 1,122.66	971.43 ± 802.46	0.64
Trauma room volume (ml)	1,664.49 ± 1,727.81	1,436.55 ± 1,327.86	0.57
Total volume (ml)	2,936.79 ± 2,562.81	2,434.78 ± 1,762.76	0.29
Time in trauma room (minutes)	58.51 ± 27.22	52.61 ± 23.28	0.03*
Preclinical rescue time (minutes)	47.35 ± 23.48	39.89 ± 16.93	0.23
Rate of whole-body CT	59.20	62.86	0.94
Multi-organ failure	33.68	32.82	0.86
Sepsis	20.40	12.38	0.03*
Intubation at accident scene	59.62	60.96	0.73
Resuscitation at accident scene	8.33	3.96	0.067
Thoracic drainage at accident scene	7.35	1.97	0.01*
Intubation in trauma room	41.10	42.13	0.77
Resuscitation in trauma room	3.48	5.15	0.41
Thoracic drainage in trauma room	13.93	12.70	0.72
Mortality rate	28.10	33.01	0.27

Data presented as mean ± standard deviation or percentage. AIS, Abbreviated Injury Scale; CCT, cranial computed tomography; CT, computed tomography; ISS, Injury Severity Score. *Significant difference.

## Discussion

The results of this study are in contrast to those of previous studies, in which female patients had better survival after trauma. In our patient cohort, the mortality rate in female patients was significantly higher than in male patients. However, women were significantly older and suffered more serious head trauma. One reason for the severe head traumas in female patients could be the cause of injury.

In initial laboratory tests the Hb levels were significantly lower in women. The volume of fluid replacement was also lower in women. However, there were no differences in systolic blood pressure and heart rate, suggesting that female patients might not have experienced more serious shock than male patients.

The ICU stay and the total hospital stay were shorter for female patients. Female patients also developed less septic events. The rate of MOF was equal between genders. This finding is consistent with results reported in a number of previous studies. Jeschke and colleagues reported a higher level of anabolic hormones in female patients, which led to a significantly shorter ICU stay. This result was explained by a decreased incidence of severe sepsis and improved wound healing. This study was performed in children who sustained a severe thermal injury [21]. Schroeder and colleagues reported a better survival rate for women after sepsis. The authors matched patient groups by age and degree of organ dysfunction. Men were observed to have decreased levels of testosterone, while testosterone levels in female patients remained normal. Estrogen was increased in both male and female patients, with women having higher levels, but this difference was not significant. Interleukin-10 was also higher in women. The mortality rate was significantly higher for men (70% vs. 26%). This difference was explained by an interaction between the immune and endocrine systems [22]. Hsieh and colleagues conducted a review of metabolic modulators following trauma sepsis. The authors reported ‘that sex steroids not only modulate the immune/cardiovascular responses but also influence various metabolic processes following trauma’ [23]. The results of these previous studies provide possible explanations for our finding that women suffered less sepsis after trauma.

According to Hussmann and colleagues an increased fluid replacement volume led to increased mortality [24]. In our analysis, women, who received a lower fluid volume, had poorer outcomes. This finding could be due the fact that women have a relatively smaller body volume than men and a higher proportion of fatty tissue, leading to a lower absolute fluid volume but a similar relative fluid volume.

In the ISS-adjusted analysis, significant gender differences decreased as ISS increased. In almost all groups, a trend to a higher mortality rate in female patients could be observed. Interestingly, the mortality rate was also higher than the RISC score, which is designed to predict mortality.

In the matched-pair analysis, in which ISS, AIS, age and the cause of trauma were similar between genders, we confirmed that women had lower Hb levels after trauma and received less fluid volume. However, the pre-hospital systolic blood pressure and the pulse rate were not different between genders. The data also demonstrated that the length of ICU stay and the rate of sepsis were higher in male patients.

No significant differences in mortality rate were observed in the matched-pair analysis. Only a trend towards a higher mortality rate in female patients was found. Women died earlier after trauma than men. However, this finding was not significantly different. It is possible that women are at higher risk for death in the early days following trauma. Another possibility is that a higher percentage of women died at the scene and did not reach the hospital. Data are not available to test this hypothesis in Germany. When female patients survived the initial trauma, they had better outcomes than male patients, with shorter stays in the ICU and in the hospital and lower rates of sepsis. To confirm these findings and to identify statistically significant differences, a matched-pair analysis with a larger patient population is necessary.

## Conclusions

In the matched-pair analysis no statistically differences were found towards the mortality rate between genders. The data suggest that women are more vulnerable in the first days after trauma. In later days, women had a better chance of survival because they had a lower sepsis rate than men. In this study, women suffered worse head trauma. This might be explained by the cause of injury. Women are more frequently involved in pedestrian accidents, whereas men are more often victims of motorcycle accidents.

## Key messages

• Women are more frequently involved in pedestrian accidents, whereas men are more often victims of motorcycle accidents.

• Women suffered worse head trauma, most probably because of the trauma mechanism.

• There is no statistically significant difference in mortality rate between genders.

• The rate of sepsis is lower in women.

• Women have a greater tendency to die soon after trauma.

## Abbreviations

AIS: Abbreviated injury scale; DGU: German society for trauma surgery; Hb: Hemoglobin; ISS: Injury severity score; MOF: Multi-organ failure; RISC: Revised injury severity classification.

## Competing interests

The authors declare that they have no competing interests.

## Authors’ contributions

CS and SL designed the study. CS, MDK, BH, DS and JK collected and analyzed the data. CS drafted the manuscript, and all authors contributed substantially to its revision. CS takes responsibility for the paper as a whole. All authors read and approved the final manuscript for publication.
